# Arthroscopic Capsular Release Versus Manipulation under Anesthesia for Refractory Frozen Shoulder: A Systematic Review with Meta‐Analysis

**DOI:** 10.1111/os.14077

**Published:** 2024-05-15

**Authors:** Yanmin Zhao, Ting Yang, Chenchen Feng, Lang Li, Long Pang, Shuzhen Zhao

**Affiliations:** ^1^ Outpatient Department, West China Hospital Sichuan University Chengdu People's Republic of China; ^2^ West China School of Nursing Sichuan University Chengdu People's Republic of China; ^3^ Operating Room of Anesthesia Surgery Center, West China Hospital Sichuan University Chengdu People's Republic of China; ^4^ Department of Orthopedics Hospital of Chengdu Office of People's Government of Tibetan Autonomous Region (Hospital.C.T.) Chengdu People's Republic of China; ^5^ Sports Medicine Center, West China Hospital Sichuan University Chengdu People's Republic of China; ^6^ Department of Orthopedics, Orthopedic Research Institute, West China Hospital Sichuan University Chengdu People's Republic of China

**Keywords:** Arthroscopic Capsular Release, Frozen Shoulder, Manipulation Under Anesthesia, Meta‐analysis, Systematic Review

## Abstract

**Objective:**

Frozen shoulder (FS) is a painful and debilitating condition affecting the shoulder joint. When patients fail to improve after conservative treatments, operative treatments including arthroscopic capsular release (ACR) and manipulation under anesthesia (MUA) are recommended. However, the comparison between these two interventions remains controversial. This study aimed to compare the efficacy and safety of ACR and MUA for refractory FS.

**Methods:**

A systematic review and meta‐analysis was conducted following the Preferred Reporting Items for Systematic Review and Meta‐analyses (PRISMA) guidelines. PubMed, EMBASE, Cochrane Library, and Web of Science were searched for eligible studies until December 10, 2023. Meta‐analyses were conducted using Manager V.5.3.3. Pooled effect sizes were expressed as the weighted mean difference (WMD) or odds ratio (OR) with 95% confidence intervals (CIs).

**Results:**

A total of eight comparative studies with 768 patients were included. Compared with MUA, ACR had statistically better Δ VAS (WMD, −0.44; 95% CI, −0.71 to −0.18; *I* ^2^ = 6%; *p* = 0.001) at over 12‐month follow‐up, which did not reach the minimal clinically important difference (MCID). Other outcomes regarding pain relief, function, and range of motion (ROM) improvements were not statistically different between the two groups at different follow‐up timepoints. Compared with the MUA group, the ACR group had a significantly higher rate of severe complications (OR, 4.14; 95% CI, 1.01 to 16.94; I2 = 0%; *p* = 0.05), but comparable rates of mild complications and additional intervention.

**Conclusions:**

In treating refractory FS, ACR demonstrated comparable pain relief, functional and ROM improvements, rates of mild complications and additional intervention but a higher risk of severe complications to MUA during short‐term follow‐up periods. Notably, ACR exhibited statistically superior improvement in the long‐term pain relief compared to the MUA group, although it did not reach the MCID.

## Graphical abstract

Arthrocopic Capsular Release Versus Manipulation under Anesthesia

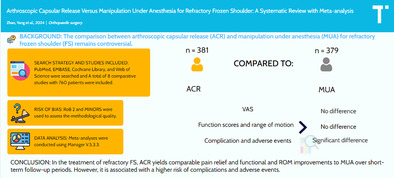

## Introduction

Frozen shoulder (FS), also known as adhesive capsulitis, is a debilitating condition characterized by pain and stiffness in the shoulder joint with a reported lifetime prevalence of 2%–5% in the general population.^1^, ^2^ Women are more frequently affected than males, with the incidence as 3.38 and 2.36 per 1000 person years, respectively.^3^ People aged between 40 and 60 are more likely to suffer from FS, with the highest incidence observed in the mid‐50s.^4^ Individuals with risk factors including cardiovascular diseases, hypothyroidism, autoimmune diseases, hypercholesterolemia, smoking, obesity, lack of physical activity, and especially diabetes mellitus are at a higher likelihood of developing FS.^5^, ^6^, ^7^, ^8^, ^9^, ^10^, ^11^, ^12^, ^13^ Compare with general population, patients with diabetes have a 10% to 20% lifetime incidence of suffering from FS and are more likely to have bilateral FS.^1^, ^14^

FS can be primary, meaning there is no specific cause, or secondary, which indicates that it is associated with another underlying condition such as trauma, surgery, or other pathology.^15^ Although the definition and classification of FS remain uncertain, it is commonly described that a typical FS progresses through three stages: freezing, frozen, and thawing, from inflammation and fibrosis within the capsule to the spontaneous resolution of this fibrosis.^16^, ^17^ The freezing stage is characterized by gradually worsening pain and progressive loss of shoulder motion. This stage can last anywhere from 6 weeks to 9 months. Following the freezing stage is the frozen stage, during which the shoulder becomes increasingly stiff, with loss of axillary recess and minimal synovitis. This stage can last from 4 to 12 months. Finally, the thawing stage involves a gradual improvement in shoulder pain and motion, which can take anywhere from 6 months to 2 years to fully resolve. While FS is normally considered to be a self‐limiting disease, the symptoms can be very extremely painful and might not disappear after the three stages.

Because the pathophysiology of FS is still poorly understood, standard evidence‐based management recommendations are absent. In general, conservative treatments, including physiotherapy, medicines, and corticosteroid injections (CSIs), are effective in up to 90% of patients with FS.^18^, ^19^, ^20^ However, in some cases, the condition may fail to improve after conservative treatments for 6 to 9 months, which would be considered refractory FS.^19^, ^21^, ^22^ Operative management, including manipulation under anesthesia (MUA) and arthroscopic capsular release (ACR), is recommended for refractory FS.^23^

MUA is recommended to performed in the second stage of FS when conservative measures have not been successful.^24^ During this procedure, the surgeon moves the patient's arm in various directions when the patient is under general or regional anesthesia. One of its key advantages is that the fibrosed capsule will be forcibly broken, allowing for improved shoulder mobility and reduced pain.^23^ However, some patients, especially those with comorbidities and older age, may not be suitable for this procedure due to the complication associated with MUA (such as fracture, dislocation, rotator cuff tear, labral tear, brachial plexus injury, and complex regional pain syndrome).^25^, ^26^, ^27^, ^28^, ^29^

Due to the advances in arthroscopic technologies, ACR has been another promising treatment for FS. This is an invasive surgical procedure that can precisely release the fibrosed capsule under direct visualization. It has been a preferred option for some surgeons to treat refractory FS because it allows a diagnostic examination and additional operations throughout the affected shoulder joint.^23^, ^30^ Current literature on the comparison between MUA and ACR is limited, and the results were conflicting.^31^, ^32^, ^33^ With some newly published articles on this topic, a high‐quality systematic review and meta‐analysis is needed.

The purpose of this study was to compare MUA and ACR in the treatment of refractory FS. It is hypothesized that the two operative treatments are equally successful in treating refractory FS, but the incidence of complications would be different.

## Methods

This systematic review was conducted according to the Preferred Reporting Items for Systematic Review and Meta‐analyses (PRISMA) statement.^34^ The study has been registered in The International Prospective Register of Systematic Reviews (PROSPERO) with the registration ID CRD42024506711.

### Search Strategy

Two independent researchers individually performed searches on PubMed, EMBASE, the Cochrane Library, and Web of Science. The searches were done using the specified search terms: (Frozen shoulder OR Adhesive capsulitis OR shoulder stiffness OR FS) AND (Manipulation OR Manipulation under anesthesia OR MUA) AND (Arthroscopic capsular release OR Surgical release of capsule OR ACR OR CR OR Lysis OR Release OR Division OR resection). There was no temporal limitation regarding the publication date. All potentially qualifying studies comparing ACR and MUA were obtained manually. Any contentious disagreement was addressed with the intervention of a third researcher.

### Selection Criteria

The inclusion criteria are as follows: (1) patients who have a confirmed diagnosis of primary FS; (2) patients in one group underwent MUA whereas patients in another group had ACR; (3) comparative studies reported in the English language; (4) at least one of the following outcomes were reported: pain, function, range of motion (ROM), complications and adverse events.

The exclusion criteria are as follows: (1) secondary FS; (2) presence of other shoulder conditions such as rotator cuff injury, instability, infection, tumor, and severe osteoarthritis; (3) patients with prior shoulder surgeries; (4) patients who did not undergo conservative treatments before MUA or ACR.

### Data Extraction

Data from the studies included in the analysis was collected independently by two researchers. All relevant information from the selected studies, including the first author, year, country, level of evidence (LOE), and sample size, as well as baseline information on the patients such as age, gender, comorbidities, symptom duration, and follow‐up time, was thoroughly recorded. The study collected and combined the following clinical outcomes: (1) change (Δ) in pain assessed using a visual analog scale (VAS); (2) Δ function scores assessed using the Constant–Murley score (CMS) and the American Shoulder and Elbow Surgeons (ASES) Standardized Shoulder Assessment Form score; (3) Δ ROM measured in degree; and (4) complications and adverse events related to the interventions. The minimal clinically important difference (MCID) was defined as a minimum of 1.4‐point change (on a 10‐point scale) in VAS,^35^ a minimum of 10‐point change in CMS and 12‐point change in ASES,^36^ and a minimum of 10° change in ROM.^36^

### Quality Assessment

Two researchers assessed the methodological quality of included studies independently, using the revised Cochrane Risk of Bias 2 (RoB 2) instrument^37^ for randomized controlled trials (RCTs) and the methodological index for non‐randomized studies (MINORS)^38^ for studies that were not RCTs. Discussions were carried out to solve any disagreements. Publication bias was not studied as the number of publications in each study field was fewer than 10, resulting in insufficient statistical power.

### Data Synthesis and Analysis

Manager V.5.3.3 (The Cochrane Collaboration, Software Update, Oxford, UK) was used for statistical assessments. To assess the outcomes, we calculated the weighted mean difference (WMD) and pooled odds ratio (OR) with corresponding 95% confidence intervals (CIs). A *P* value no greater than 0.05 was considered statistically significant. We analyzed the heterogeneity of each qualifying trial using Cochrane's *Q* and *I* ^*2*^ statistics. Heterogeneity is inevitable due to the influence of race, age, intervention, and other factors in the included studies. Therefore, random‐effects models were used for this meta‐analysis, regardless of the *I* ^*2*^. To assess potential publication biases, funnel plots summarizing all outcomes were examined.

## Results

### Search Results and Study Characteristics

**FIGURE 1:**
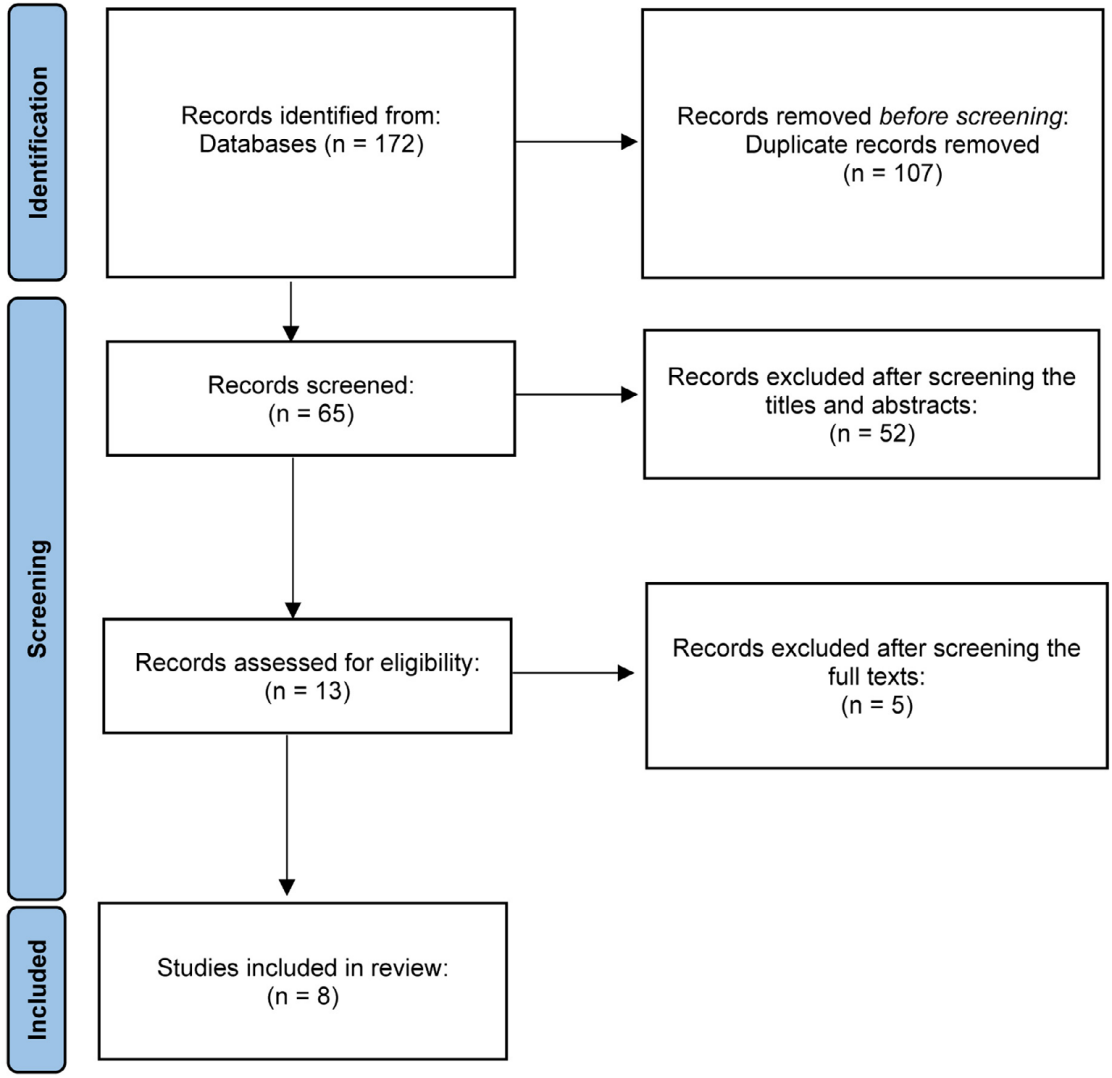
Flow chart of literature retrieval.

After searches on PubMed, EMBASE, the Cochrane Library, and Web of Science according to the search terms by two independent researchers, a total of 172 available articles until December 10, 2023 were retrieved. After removing 107 duplicates, the same two researchers screened the titles and abstracts of the remaining articles, and 52 articles were excluded. Then, the same two researchers screened the full texts and references of the remaining 13 articles. Finally, five studies^39^, ^40^, ^41^, ^42^, ^43^ were excluded according to the selection criteria, and eight studies^44^, ^45^, ^46^, ^47^, ^48^, ^49^, ^50^, ^51^ were included in this systematic review and meta‐analysis (Figure 1). The detailed characteristics of the enrolled studies are shown in Table 1.

**TABLE 1:** Characteristics of the included studies.

First author	Year	Country	LOE	Patients (shoulders), n		Age, years		Gender (M/F)		Diabetes, %		Time of symptoms, m		Follow‐up period, m	
						Mean ± SD (Range)									
														Mean ± SD (Range)	
												Mean ± SD (Range)			
				ACR	MUA	ACR	MUA	ACR	MUA	ACR	MUA	ACR	MUA	ACR	MUA
Rangan	2020	UK	I	191 (191)	189 (189)	54.4 ± 7.6	54.4 ± 7.3	74/117	68/121	29%	31%	11.3 ± 10.1	10.7 ± 8.7	12 ± −	
Inclusion	(1) 18 years or older; (2) clinical diagnosis of unilateral frozen shoulder, characterized by the restriction of passive external rotation in the affected shoulder to less than 50% of the opposite shoulder.														
Exclusion	(1) Patients with bilateral concurrent frozen shoulder; (2) frozen shoulder secondary to trauma that required hospital care or secondary to other causes, except diabetes; (3) not having sufficient mental capacity to understand the instructions or treatment.														
Kim	2020	Korea	III	30 (30)	30 (30)	55.3 ± 8.3	54.5 ± 7.7	9/21	9/21	43.3%	36.7%	11.4 ± 8.3	12.1 ± 8.7	12 ± −	
Inclusion	(1) Patients with the diagnosis of frozen shoulder, defined as limitation of motion by greater than 50% in at least two planes (compared to the unaffected shoulder); (2) absence of intrinsic or extrinsic shoulder disease confirmed by MRI or ultrasonography; (3) unsuccessful nonoperative management (e.g., medications, steroid injections, or physical therapy) for at least 3 months.														
Exclusion	(1) Secondary shoulder stiffness with a rotator cuff tear, calcific tendinitis, osteoarthritis, inflammatory arthritis, and postsurgical, posttraumatic, or cervical disc disorder.														
Lee	2020	Korea	III	22 (22)	57 (57)	53.9 ± 6.4	55.3 ± 8.5	8/14	24/33	31.8%	29.8%	6.6 ± 4.1	6.4 ± 3.7	7.2 ± 1.6	7.7 ± 1.7
Inclusion	(1) Patients with the diagnosis of frozen shoulder characterized by functional restriction of both active and passive shoulder motion for which radiographs of the glenohumeral joint were essentially unremarkable except for the possible presence of osteopenia; (2) refractory to conservative treatment (intra‐articular steroid injections and physical therapy) for at least 6 months; (3) restriction of both passive and active glenohumeral and scapulothoracic motion of equal to or less than 100° of elevation and less than 50% of external rotation, as compared to the contralateral side.														
Exclusion	(1) Patients with rotator cuff tear, shoulder osteoarthritis, calcified tendinitis confirmed by MRI; (2) hemiplegia after stroke, bone metastasis in the shoulder region; (3) history of shoulder fractures, and shoulder surgeries.														
Schoch	2020	USA	III	44 (44)	15 (15)	58 ± − (31–79)	59 ± − (36–80)	12/32	3/12	29.5%	20%	NA^a^	NA^a^	5.8 ± − (2–35)	9.9 ± − (2–43)
Inclusion	(1) Primary diagnosis of adhesive capsulitis or frozen shoulder.														
Exclusion	(1) Shoulders with previous surgery or requiring additional procedures at the time of intervention (rotator cuff repair, labral repair, or biceps tenotomy/tenodesis) were eliminated.														
ULUYARDIMCI	2020	Turkey	III	15 (15)	17 (17)	55.7 ± 7.2	56.1 ± 6.8	4/11	4/13	40%	35.3%	14.1 ± 6.2	13.6 ± 4.3	16.9 ± 9.1	17.3 ± 9.8
Inclusion	(1) Patients with refractory frozen shoulder defined as recalcitrant to nonoperative management (oral medications, physical therapy, and injections) for 6 months and cases with restriction in the shoulder range of motion compared with the contralateral side.														
Exclusion	(1) Patients with rotator cuff tear, calcific tendinitis, shoulder osteoarthritis, posttraumatic or postsurgical etiology; (2) patients with incomplete patient records, and less than 6 months of follow‐up; (3) patients whose shoulder functions were affected due to a musculoskeletal or neurological disease.														
Aziz	2022	Egypt	II	15 (15)	15 (15)	NA^a^ (40–80)	NA^a^ (40–80)	NA^a^	NA^a^	53.5%	46.7%	5.8 ± 1.7	4.7 ± 1.6	8.3 ± 1.7	7.8 ± 2.1
Inclusion	(1) Patients with primary frozen shoulder clinically and radiologically; (2) Age between 40 and 80 years old; 2 (3) At least 3‐months history of pain and stiffness of the shoulder; (4) Documented restriction of both passive and active glenohumeral and of equal to or less than 100° of elevation, and less than 50% of external rotation, as compared to the contralateral side.														
Exclusion	(1) patients with a history of cancer, or rheumatic disease; (2) patients with a history of surgery or suffered trauma; (3) patients with severe neurological deficit of the involved upper extremity; (4) patientswho were lost to follow‐up or did not complete the follow‐up period; (5) patients undergoing any concomitant procedure in addition to capsular release.														
Sundararajan	2022	India	I	44 (44)	41 (41)	54.2 ± 5.9	53.3 ± 6.7	NA^a^	NA^a^	65.9%	82.9%	6.3 ± 4.4	8 ± 6.4	6 ± −	
Inclusion	(1) Painfully restricted passive ROM of the shoulder (forward elevation <1000 and external rotation <50% of the opposite shoulder); (2) conservatively managed at least for 3 months with MRI findings suggestive of frozen shoulder.														
Exclusion	(1) Patients with glenohumeral arthritis, full‐thickness rotator cuff tears, history of fall/trauma, previous surgery around the shoulder joint.														
Ari	2023	Turkey	II	18 (28)	25 (25)	53.2 ± 11.5	59.3 ± 11.1	11/7	17/8	NA^a^	NA^a^	9.4 ± 2.1	8.2 ± 1.6	16.9 ± 3.1	16.6 ± 3.7
Inclusion	(1) Age ≥ 20 years; (2) repairable full‐thickness tears of the supraspinatus and/or infraspinatus diagnosed by intraoperative arthroscopy.														
Exclusion	(1) Displaced fractures or dislocations around the shoulder girdle; (2) a history of shoulder surgery; (3) inflammatory or neuropathic arthritis; (4) irreparable rotator cuff tears.														

Abbreviations: ACR, arthroscopic capsular release; F, female; LOE, level of evidence; M, male; MRI, magnetic resonance imaging; MUA, manipulation under anesthesia; NA, not applicable; UK, the United Kingdom.

^a^ Not reported.

### Risk of Bias in Included Studies

**FIGURE 2:**
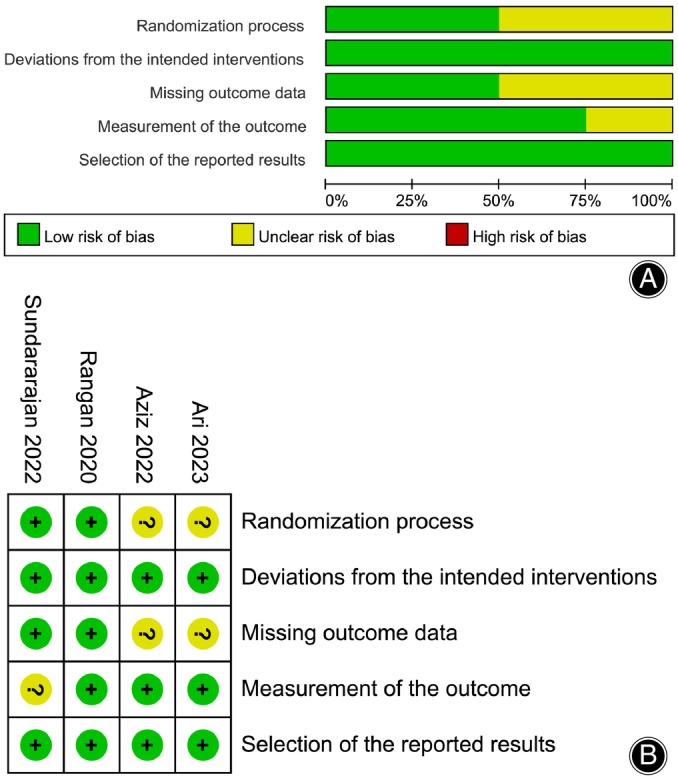
Risk of bias graph. (A) Graph of the risk of bias summary for the included RCTs; (B) graph of the risk of bias for each included RCT.

**FIGURE 3:**
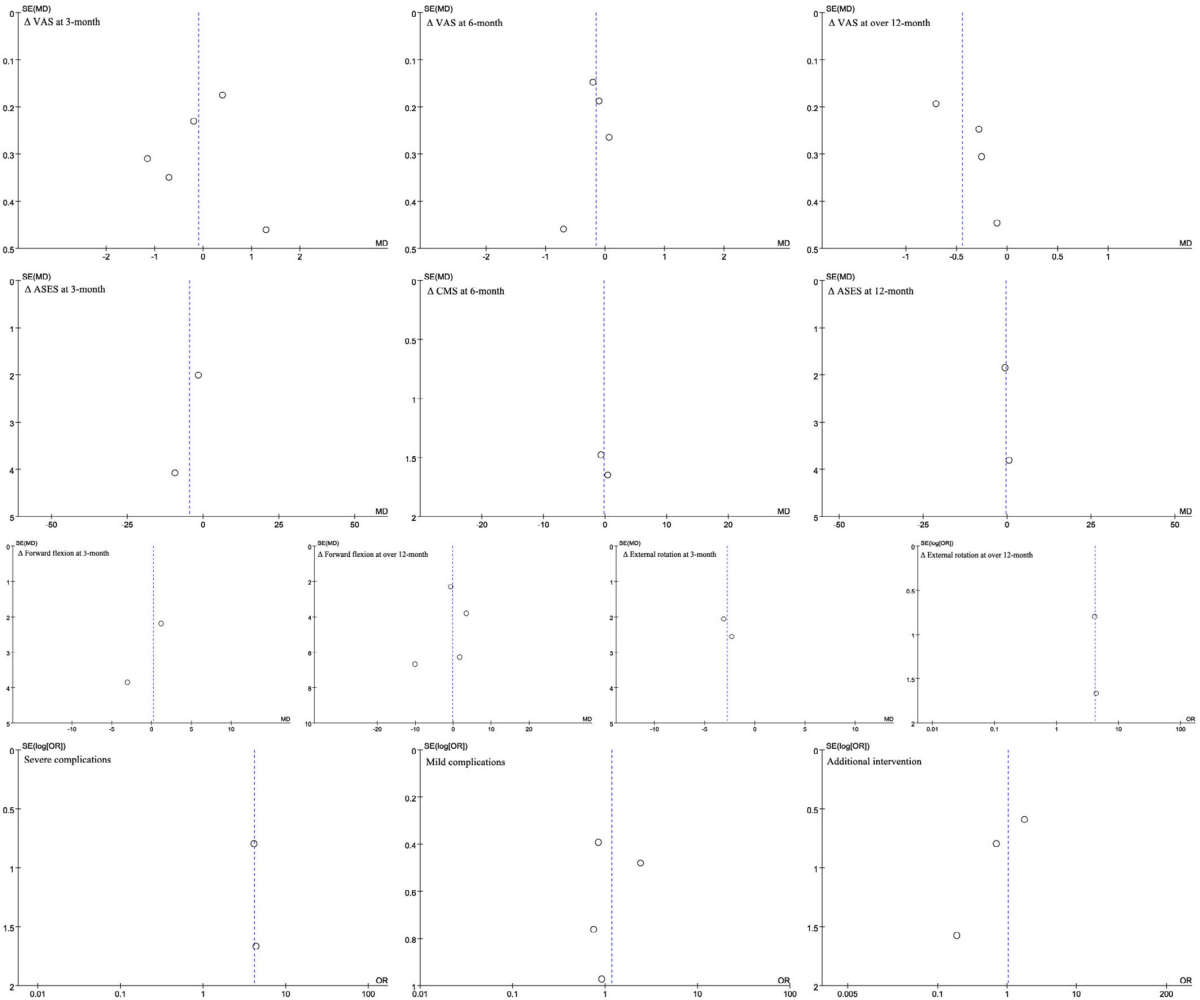
Funnel plots of all outcomes.

The risk of bias in the included RCTs was presented in Figure 2. Table 2 summarizes the risk of bias in the non‐RCT studies. Regarding publication biases, a visual inspection of the funnel plot indicated a relatively low risk (Figure 3).

**TABLE 2:** Quality assessment of the non‐RCT studies using the Methodological Index for Non‐Randomized Studies (MINORS) criteria

First author	Year	Study design	LOE	1	2	3	4	5	6	7	8	Total
Kim	2020	RCS	III	2	2	2	2	1	2	1	0	12
Lee	2020	RCS	III	2	2	2	2	1	2	1	0	12
Schoch	2020	RCS	III	2	2	0	2	1	2	1	0	10
ULUYARDIMCI	2020	RCS	III	2	2	0	2	1	2	1	0	10

*Note*: The criteria of MINORS with 0 points when not reported, 1 when reported but not adequate, and 2 when reported and adequate. Maximum score is 16. 1. A clearly stated aim; 2. Inclusion of consecutive patients; 3. Prospective collection of data; 4. End points appropriate to the aim of the study; 5. Unbiased assessment of the study end point; 6. Follow‐up period appropriate to the aim of the study; 7. Loss to follow‐up less than 5%; 8. Prospective calculation of the study size.

Abbreviations: LOE, level of evidence; RCS, retrospective cohort study.

### Pain

**FIGURE 4:**
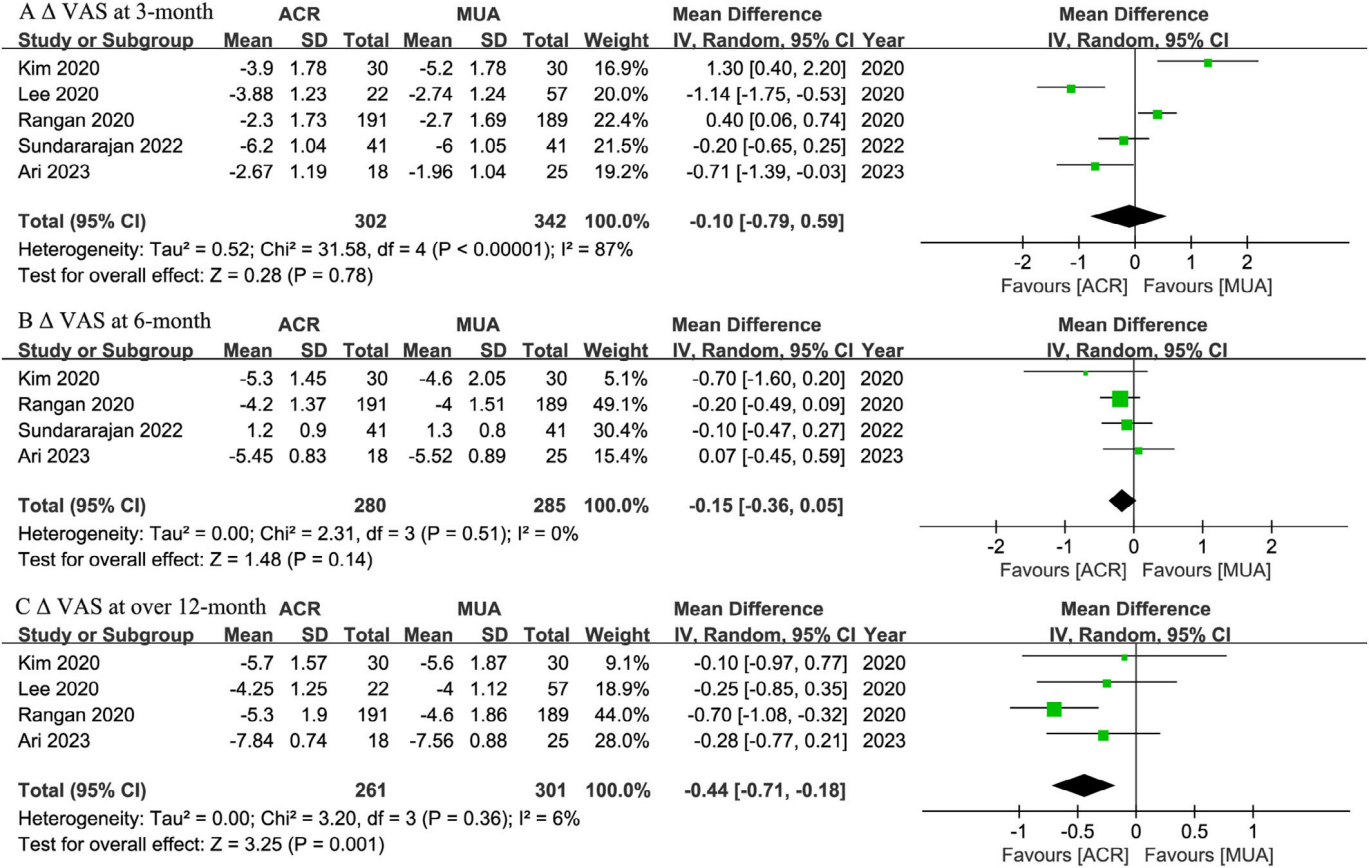
Meta‐analysis of ACR versus MUA: Δ VAS at 3‐month, 6‐month, and over 12‐month follow‐ups. The green squares represent the effect estimate of the individual studies and the horizontal lines indicate the confidence interval, and the dimension of the square reflects the weight of each study. The black diamond represents the combined point estimate and confidence intervals.

Five,^44^, ^46^, ^47^, ^48^, ^50^ four,^44^, ^46^, ^48^, ^50^ and four^44^, ^46^, ^47^, ^48^ trials, respectively, provided postoperative 3‐month, 6‐month, and at least 12‐month VAS results (Figure 4). No significant differences in Δ VAS were identified between the ACR and MUA groups at 3‐month (WMD, −0.10; 95% CI, −0.79 to 0.59; *I* ^*2*^ = 87%; *p* = 0.78) and 6‐month (WMD, −0.15; 95% CI, −0.36 to 0.05; *I* ^*2*^ = 0%; *p* = 0.14) follow‐ups. At over 12‐month follow‐up, the ACR group had statistically better Δ VAS than the MUA group (WMD, −0.44; 95% CI, −0.71 to −0.18; *I* ^*2*^ = 6%; *p* = 0.001). However, this difference did not reach the MCID for VAS. Subgroup analyses were presented in Table 3.

**TABLE 3:** Summary of outcomes and subgroup analyses.

Study design	No. of studies (patients)	WMD	95% CI	*I* ^*2*^	*p* value	Comparison	MCID reached
Δ VAS at 3‐month follow‐up							
Overall	5 (644)	−0.10	−0.79, 0.59	87	0.78	ACR = MUA	Not applicable
RCT	3 (505)	−0.12	−0.73, 0.49	80	0.71	ACR = MUA	Not applicable
Non‐RCT	2 (139)	0.06	−2.33, 2.45	95	0.96	ACR = MUA	Not applicable
Δ VAS at 6‐month follow‐up							
Overall	4 (565)	−0.15	−0.36, 0.05	0	0.14	ACR = MUA	Not applicable
RCT	3 (505)	0.06	−0.33, 0.08	0	0.24	ACR = MUA	Not applicable
Non‐RCT	1 (60)	−0.70	−1.60, 0.20	Not applicable	0.13	ACR = MUA	Not applicable
Δ VAS at ≥12‐month follow‐up							
Overall	4 (562)	−0.44	−0.71, −0.18	6	0.001	ACR > MUA	No
RCT	2 (423)	−0.52	−0.93, −0.11	44	0.01	ACR > MUA	No
Non‐RCT	2 (139)	−0.20	−0.70, 0.29	0	0.42	ACR = MUA	Not applicable
Δ ASES at 3‐month follow‐up							
Overall	2 (139)	−4.60	−12.08, 2.88	66	0.23	ACR = MUA	Not applicable
RCT	0 (0)	Not applicable	Not applicable	Not applicable	Not applicable	Not applicable	Not applicable
Non‐RCT	2 (139)	−4.60	−12.08, 2.88	66	0.23	ACR = MUA	Not applicable
Δ CMS at 6‐month follow‐up							
Overall	2 (125)	−0.21	−2.36, 1.94	0	0.85	ACR = MUA	Not applicable
RCT	2 (125)	−0.21	−2.36, 1.94	0	0.85	ACR = MUA	Not applicable
Non‐RCT	0 (0)	Not applicable	Not applicable	Not applicable	Not applicable	Not applicable	Not applicable
Δ ASES at ≥12‐month follow‐up							
Overall	2 (139)	−0.39	−3.66. 2.88	0	0.82	ACR = MUA	Not applicable
RCT	0 (0)	Not applicable	Not applicable	Not applicable	Not applicable	Not applicable	Not applicable
Non‐RCT	2 (139)	−0.39	−3.66. 2.88	0	0.82	ACR = MUA	Not applicable
Δ Forward flexion at 3‐month follow‐up							
Overall	2 (139)	0.17	−3.55, 3.90	0	0.93	ACR = MUA	Not applicable
RCT	0 (0)	Not applicable	Not applicable	Not applicable	Not applicable	Not applicable	Not applicable
Non‐RCT	2 (139)	0.17	−3.55, 3.90	0	0.93	ACR = MUA	Not applicable
Δ Forward flexion at ≥12‐month follow‐up							
Overall	4 (230)	−0.25	−4.05, 3.55	6	0.90	ACR = MUA	Not applicable
RCT	0 (0)	Not applicable	Not applicable	Not applicable	Not applicable	Not applicable	Not applicable
Non‐RCT	4 (230)	−0.25	−4.05, 3.55	6	0.90	ACR = MUA	Not applicable
Δ External rotation at 3‐month follow‐up							
Overall	2 (139)	−2.79	−5.93, 0.35	0	0.08	ACR = MUA	Not applicable
RCT	0 (0)	Not applicable	Not applicable	Not applicable	Not applicable	Not applicable	Not applicable
Non‐RCT	2 (139)	−2.79	−5.93, 0.35	0	0.08	ACR = MUA	Not applicable
Δ External rotation at ≥12‐month follow‐up							
Overall	4 (230)	−0.44	−4.12, 3.24	42	0.81	ACR = MUA	Not applicable
RCT	0 (0)	Not applicable	Not applicable	Not applicable	Not applicable	Not applicable	Not applicable
Non‐RCT	4 (230)	−0.44	−4.12, 3.24	42	0.81	ACR = MUA	Not applicable
Severe complications							
Overall	8 (768)	4.14	1.01, 16.94	0	0.05	MUA > ACR	Not applicable
RCT	2 (423)	4.14	1.01, 16.94	0	0.05	MUA > ACR	Not applicable
Non‐RCT	6 (345)	Not estimate	Not estimate	Not applicable	Not applicable	Not applicable	Not applicable
Mild complications							
Overall	8 (768)	1.18	0.66, 2.09	10	0.58	ACR = MUA	Not applicable
RCT	4 (538)	1.18	0.66, 2.09	10	0.58	ACR = MUA	Not applicable
Non‐RCT	4 (230)	Not estimate	Not estimate	Not applicable	Not applicable	Not applicable	Not applicable
Additional intervention							
Overall	8 (775)	1.03	0.38, 2.75	12	0.96	ACR = MUA	Not applicable
RCT	4 (545)	0.70	0.15, 3.31	Not applicable	0.65	ACR = MUA	Not applicable
Non‐RCT	4 (230)	0.91	0.12, 6.97	46	0.92	ACR = MUA	Not applicable

Abbreviations: ACR, arthroscopic capsular release; CI, confidence interval; MCID, minimal clinically important difference; MUA, manipulation under anesthesia; RCT, randomized controlled trial; WMD, weighted mean difference.

### Function Scores

**FIGURE 5:**
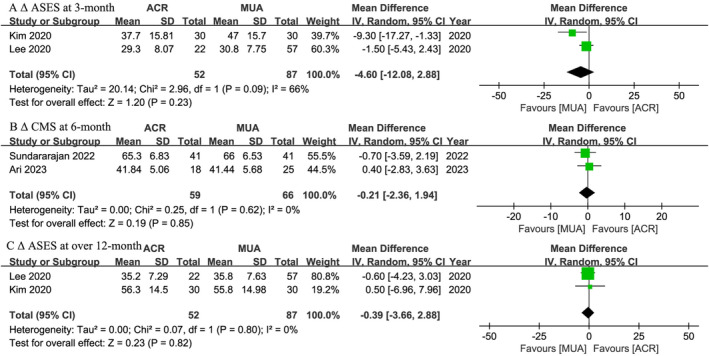
Meta‐analysis of ACR versus MUA: Δ ASES at 3‐month and over 12‐month follow‐ups; Δ CMS at 6‐month follow‐up. The green squares represent the effect estimate of the individual studies and the horizontal lines indicate the confidence interval, and the dimension of the square reflects the weight of each study. The black diamond represents the combined point estimate and confidence intervals.

Two studies^46^, ^47^ reported ASES results, and there was no statistically significant difference in Δ ASES at either 3‐month (WMD, −4.60; 95% CI, −12.80 to 2.88; *I* ^*2*^ = 66%; *p* = 0.23) or over 12‐month (WMD, −0.39; 95% CI, −3.66 to 2.88; *I* ^*2*^ = 0%; *p* = 0.82) follow‐ups. Another two studies^44^, ^50^ found that the two groups had similar Δ CMS at 6‐month follow‐up (WMD, −0.21; 95% CI, −2.36 to 1.94; *I* ^*2*^ = 0%; *p* = 0.85) (Figure 5). Subgroup analyses were presented in Table 3.

### Range of Motion

**FIGURE 6:**
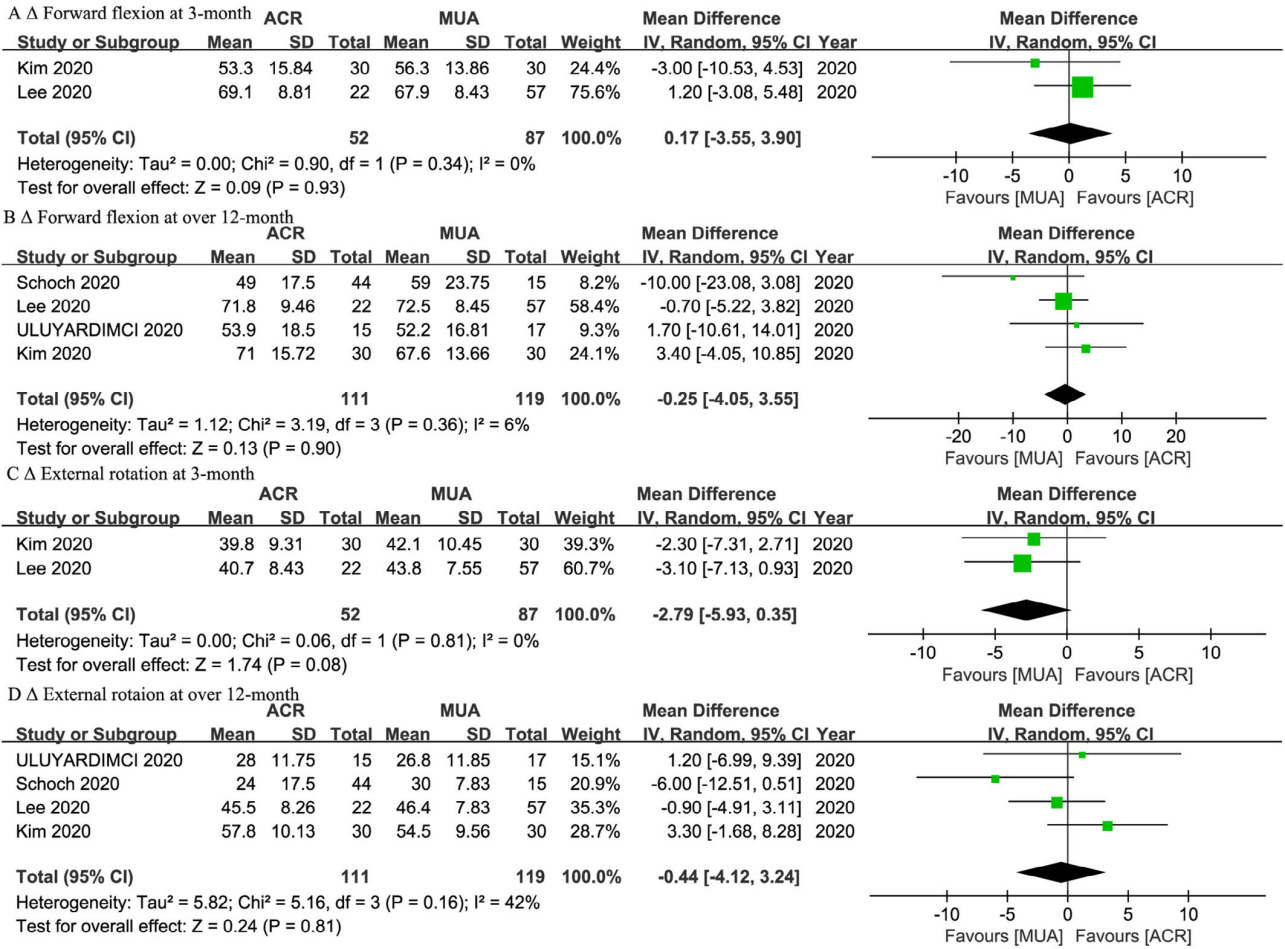
Meta‐analysis of ACR versus MUA: Δ forward flexion and Δ external rotation at 3‐month and over 12‐month follow‐up. The green squares represent the effect estimate of the individual studies and the horizontal lines indicate the confidence interval, and the dimension of the square reflects the weight of each study. The black diamond represents the combined point estimate and confidence intervals.

Two studies^46^, ^47^ reported similar Δ forward flexion (WMD, 0.17; 95% CI, −3.55 to 3.90; *I* ^*2*^ = 0%; *p* = 0.93) and Δ external rotation (WMD, −2.79; 95% CI, −5.93 to 0.35; *I* ^*2*^ = 0%; *p* = 0.08) between two groups at 3‐month follow‐up. Four studies^46^, ^47^, ^49^, ^51^ reported similar Δ forward flexion (WMD, −0.25; 95% CI, −4.05 to 3.55; *I* ^*2*^ = 6%; *p* = 0.90) and Δ external rotation (WMD, −0.44; 95% CI, −4.12 to 3.24; *I* ^*2*^ = 42%; *p* = 0.81) between two groups at over 12‐month follow‐up (Figure 6). Subgroup analyses were presented in Table 3.

### Complications and Additional Intervention

**FIGURE 7:**
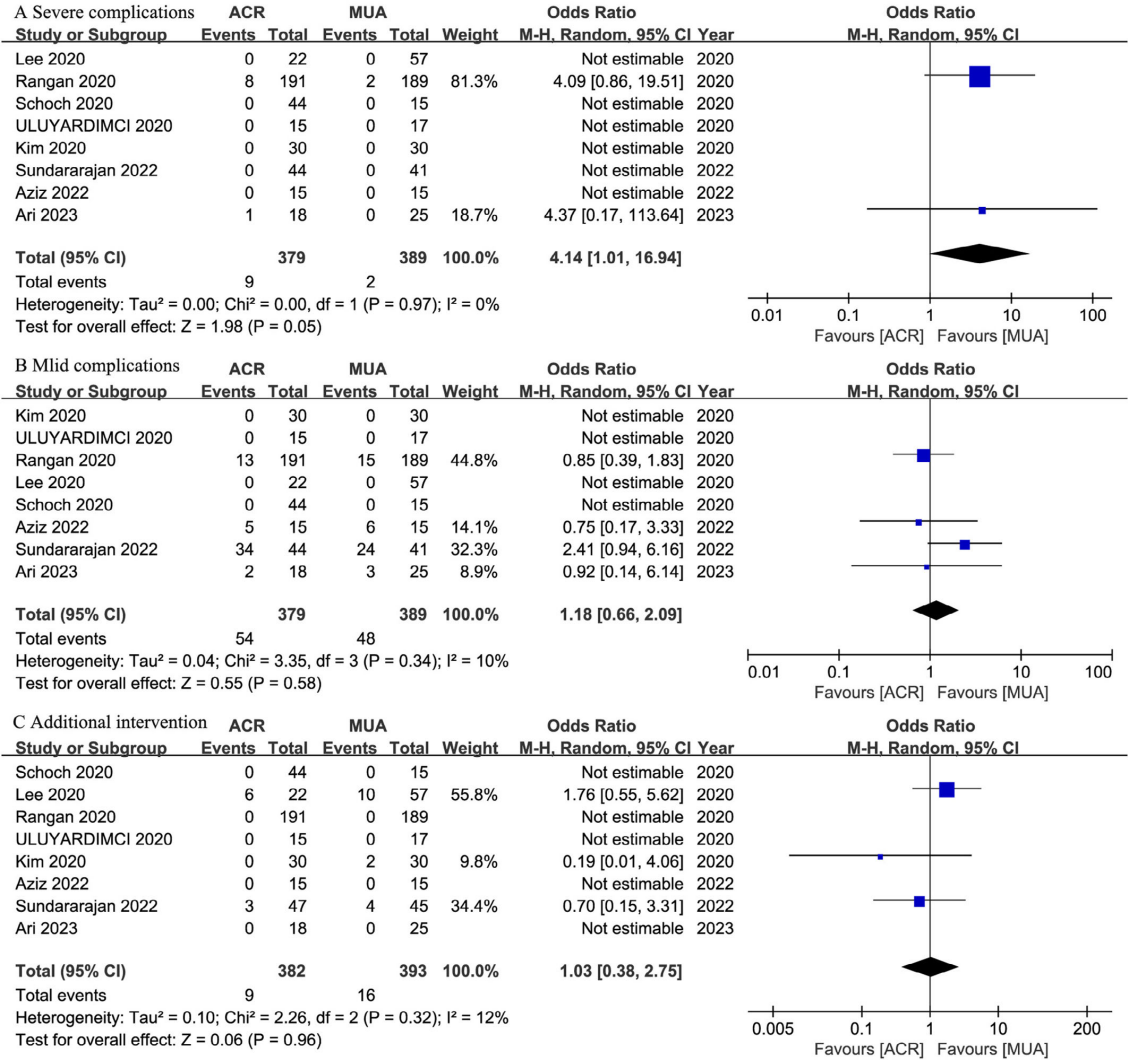
Meta‐analysis of ACR versus MUA: postoperative complications and additional intervention.

All studies^44^, ^45^, ^46^, ^47^, ^48^, ^49^, ^50^, ^51^ reported postoperative complications and additional intervention related to the treatments. Severe complications were characterized as events related to the operation that exert a substantial impact on patients, typically necessitating additional medical interventions.^52^ Examples include fractures, infections, nerve injuries, deltoid detachments, and so on. Compared with the MUA group (2/389, 0.51%), the ACR group (9/379, 2.37%) had a significantly higher rate of severe complications (OR,4.14; 95% CI, 1.01 to 16.94; *I* ^*2*^ = 0%; *p* = 0.05). Mild complications were defined as events associated with the operation that result in discomfort, inconvenience, or temporary limitations, typically resolving over time or with minimal intervention.^52^ Examples include swelling, bruising, irritation around the incision site, and accumulation of fluid under the skin, among others. The ACR group (54/379, 14.24%) and the MUA group (48/389, 12.33%) showed similar rates of mild complications (OR, 1.18; 95% CI, 0.66 to 2.09; *I* ^*2*^ = 10%; *p* = 0.58). Additional intervention refers to postoperative interventions, most commonly involving intra‐articular steroid injections or a second ACR or MUA.^52^ In the ACR group, nine out of 382 (2.36%) individuals experienced additional intervention, while in the MUA group, 16 out of 393 (4.07%) required the same, with all interventions being intra‐articular steroid injections. No statistically significant difference was found between the groups regarding additional intervention (OR, 1.03; 95% CI, 0.38 to 2.75; *I* ^*2*^ = 12%; *p* = 0.96) (Figure 7). Subgroup analyses were presented in Table 3.

## Discussion

With 768 patients involved, this systematic review and meta‐analysis included eight comparative studies (four RCTs and four retrospective cohort studies) that directly compared the efficacy and safety of ACR and MUA for patients with refractory FS. The results demonstrated that there were no statistically significant differences in improvements in short‐term to medium‐term VAS scores, function scores, and ROM at all time points between the MUA and ACR groups. Furthermore, the ACR group had a significantly higher rate of severe complications, but similar rates of mild complications and additional intervention compared to the MUA group. Although the ACR group had a statistically superior improvement in the long‐term VAS score compared to the MUA group, the difference did not reach the MCID.

It is worth noting that, compared to the ACR group, a higher but not statistically different rate of additional intra‐articular steroid injection was observed in the MUA group. This suggests that, in a longer follow‐up period, the MUA group may experience more recurrences and potential steroid‐related negative effects.^53^ The pooled data suggests that ACR fails to provide clinical superiority over MUA for the treatment of refractory FS. However, given the long‐term trend in favor of ACR and the limited follow‐up durations of the included studies, we emphasize the importance of further in‐depth studies and continued research to comprehensively assess the long‐term efficacy. The existing literature reports conflicting results about the comparison of ACR and MUA for refractory FS. Therefore, there is no consensus on the choice of operative treatments when patients fail to improve after conservative treatments. The most recent systematic review on this topic dates to 10 years ago. Grant et al. included 22 studies (21 of which were level IV evidence) in this systematic review, and they suggested a mild benefit of ACR over MUA in patients with refractory idiopathic or diabetic FS.^31^ However, the quality of the evidence is low, and there was a great lack of direct comparison of the two operative treatments until the last 3 years. Only a small number of studies reported that ACR would provide more benefits than MUA when treating refractory FS,^44^, ^45^ while most studies indicated that both ACR and MUA can achieve similarly successful outcomes without major complications or adverse events.^46^, ^47^, ^48^, ^49^, ^50^, ^51^

Complications after ACR typically stem from the surgical procedure itself, such as infection, intra‐articular bleeding, nerve injuries like those affecting the axillary or musculocutaneous nerves, and infrequent glenoid fractures. Conversely, MUA complications often arise during the nerve root block or terminal range of movement, such as anesthetic‐related reactions, fracture, dislocation, brachial plexus injury, rotator cuff tear, and labral tear.^23^ In clinical practice, most complications are mild with a good prognosis, typically resolving over time or with minimal intervention such as medications, physiotherapy, and patient education.^54^ In our study, most reported complications in both groups were mild and did not require special intervention. Severe complications occurred at a rare rate and were mainly associated with structural injuries. Once proper interventions aimed at restoring the structural injuries are performed, good outcomes can also be achieved.^23^, ^28^ In our study, two cases in the ACR group and nine cases in the MUA group who experienced severe complications all had a good recovery after prompt interventions.

This present systematic review and meta‐analysis is the first study to include studies that directly compare the two interventions. With a larger sample size, this study verified that there is no significant difference in clinical outcomes between ACR and MUA and revealed that ACR had a higher risk of complications and adverse events. The conclusion is similar to that of UK FROST,^48^ the largest multicenter RCT in FS. With 503 participants recruited across 35 UK hospitals, ACR, MUA, and early structured physiotherapy with intra‐articular corticosteroid injection were compared. The researchers found that none of the three options were clinically superior, but ACR had higher risks (3.9% of individuals in this cohort experienced a significant adverse event compared to 1% of those who received MUA). In this study, the ACR group had a significantly higher rate of severe complications (2.37% vs. 0.51%, *p* = 0.05) but a similar rate of mild complications (14.24% vs. 12.33%, *p* = 0.58) when compared to the MUA group. This finding underscores the necessity for a comprehensive risk–benefit assessment when considering these two interventions, especially in the context of vulnerable patient populations with comorbidities or older age.

The study has some limitations. First, although this study only includes directly comparative studies, the number of RCTs and the sample size are still limited. Second, only two included studies had mean follow‐up periods longer than 12 months, so the long‐term outcomes are still absent. High‐quality studies with long‐term follow‐up are needed. Third, while the baseline data of each enrolled study were matched, variations in patient characteristics, surgical techniques, and rehabilitation procedures across studies were inevitable. These differences may have contributed to a relatively high heterogeneity and influenced the outcomes. Additionally, we have further delved into potential biases within the included studies. Despite our efforts to minimize bias through rigorous study selection and data extraction, we recognize that studies with statistically significant or positive results may be more likely to be published. This could introduce a bias in favor of positive outcomes. To assess this, we have included a funnel plot for all outcomes (see Figure 3) to provide transparency on the potential influence of publication bias. Variations in study designs, patient populations, and methodologies across the included studies may also introduce biases that could impact the interpretation of our findings. Finally, we only included articles written in English, which could potentially lead to the absence of studies that used different languages.

## Conclusion

In treating refractory FS, ACR demonstrated comparable pain relief, functional and ROM improvements, rates of mild complications and additional intervention but a higher risk of severe complications to MUA during short‐term follow‐up periods. Notably, ACR exhibited statistically superior improvement in the long‐term pain relief compared to the MUA group, although it did not reach the MCID. The existing evidence is constrained by limited quality, small sample sizes, and short follow‐up durations. To establish more robust conclusions, it is imperative to conduct high‐quality RCTs with long‐term follow‐up directly comparing ACR and MUA.

## Author Contributions

Conceptualization, S.Z. and C.F.; methodology, Y.M. and T.Y.; software, Y.M. and T.Y.; formal analysis, Y.M. and T.Y.; data curation, Y.M. and T.Y.; writing—original draft preparation, Y.M. and T.Y.; writing—review and editing, L.L, L.P., S.Z, and C.F.; visualization, Y.M. and T.Y.; supervision, S.Z. and C.F.

## Funding Information

L.L. and L.P. received funding from Tibet Autonomous Region Science and Technology Plan Joint Funding Project (XZ202201ZY0041G).
